# Impact of an active lifestyle on heart rate variability and oxidative stress markers in offspring of hypertensives

**DOI:** 10.1038/s41598-020-69104-w

**Published:** 2020-07-24

**Authors:** F. A. Santa-Rosa, G. L. Shimojo, D. S. Dias, A. Viana, F. C. Lanza, M. C. Irigoyen, K. De Angelis

**Affiliations:** 1 Laboratory of Translational Physiology, Universidade Nove de Julho (UNINOVE), São Paulo, Brazil; 2 Faculdade Estácio de Carapicuíba, São Paulo, Brazil; 3 School of Physical Education of Military Police of State of Sao Paulo, São Paulo, Brazil; 4 Estácio de Sá University, São Paulo, Brazil; 5 Heart Institute (InCor), Medical School, University of São Paulo, São Paulo, Brazil; 6 Physiology Department, Federal University of São Paulo, Rua Botucatu, 862, São Paulo, SP 04023 061 Brazil

**Keywords:** Lifestyle modification, Risk factors

## Abstract

Familial history of hypertension is associated with autonomic dysfunction and increase in blood pressure (BP). However, an active lifestyle has been found to improve a number of health outcomes and reduce all-cause mortality. The aim of the present study was to investigate the effects of an active lifestyle on hemodynamics, heart rate variability (HRV) and oxidative stress markers in offspring of hypertensive parents. One hundred twenty-seven subjects were assigned into four groups: sedentary offspring of normotensives (S-ON) or hypertensives (S-OH); and physically active offspring of normotensives (A-ON) or hypertensives (A-OH). Diastolic BP and heart rate were reduced in the physically active groups when compared to S-OH group. A-ON and A-OH groups presented increased values of RR total variance when compared to the sedentary ones (A-ON: 4,912 ± 538 vs. S-ON: 2,354 ± 159; A-OH: 3,112 ± 236 vs. S-OH: 2,232 ± 241 ms^2^). Cardiac sympato-vagal balance (LF/HF), systemic hydrogen peroxide and superoxide anion were markedly increased in S-OH group when compared to all other studied groups. Additionally, important correlations were observed between LF/HF with diastolic BP (r = 0.30) and hydrogen peroxide (r = 0.41). Thus, our findings seem to confirm an early autonomic dysfunction in offspring of hypertensive parents, which was associated with a systemic increase in reactive oxygen species and blood pressure. However, our most important finding lies in the attenuation of such disorders in offspring of physically active hypertensives, thus emphasizing the importance of a physically active lifestyle in the prevention of early disorders that may be associated with onset of hypertension.

## Introduction

Hypertension accounts for 13.5 million deaths worldwide every year and for about half the global risk for stroke and ischemic heart disease^1^. In the US alone, 77.9 million adults have high blood pressure (BP), and by 2025 more than 500 million people may be affected^2^. The heritable component of BP has been documented in family history, suggesting that 30%–50% of the increase in BP may be attributed to genetic heritability and around 50% to environmental factors^3^. Although family history is an important non-modifiable risk factor for hypertension onset, this substantial heritability has prompted extensive efforts to identify its genetic underpinnings^4-6^. A meta-analysis has identified several genome-wide significant associations with hemodynamic parameters and hypertension^4^. Previous studies have reported that genetic variability of some of the sympathetic nervous system (SNS) genes encoding catecholamine metabolism, transport, receptors, and signal transduction play a critical role in the onset of essential hypertension and organ damage^7^. These findings indicate that both the genetic component and the SNS may play a critical role in hypertension development in offspring of hypertensives. In this sense, we have recently observed that autonomic control of circulation is the first mechanism affected by chronic fructose consumption initiated after weaning in spontaneously hypertensive rats (SHR), followed by unfavorable systemic changes in inflammatory and oxidative stress markers, leading to a later exacerbated increase in blood pressure^8^.

In fact, neuronal networks are effective mechanisms selected by evolution to control physiological homeostasis^9^. Heart rate variability (HRV) assessed by power spectral analysis is a great tool to obtain reliable indices of overall autonomic nervous system modulation and baroreceptor function^10^. The control of the BP by SNS has been studied for years, and it may well be one of the underlying mechanisms of increased BP in offspring of hypertensives^7,8^. Additionally, oxidative stress has been found to play an important role in the development of hypertension and cardiovascular diseases^9,10^. Previous studies have demonstrated a positive correlation between sympathetic modulation and oxidative stress in experimental models^11,12^. These findings suggest that the SNS may act as a key trigger to oxidative stress in the onset of hypertension. However, the evidence for this relationship in humans is yet to be fully understood.

On the other hand, the effective contribution of an active lifestyle to improved health outcomes and decreased all-cause mortality has been widely acknowledged^13^. Previous studies have reported the positive effects of an active lifestyle on autonomic cardiac modulation of hypertensives and parental hypertension^14,15^. However, the mechanisms underlying the role of an active lifestyle in improving vascular health, BP and oxidative stress remain unclear. Therefore, we hypothesized that a dysfunction in the cardiac autonomic modulation plays a critical role in inducing oxidative stress and an active lifestyle may indeed be an effective approach to manage these early dysfunctions and control of BP in offspring of hypertensive parents. To address this issue, the purpose of the present study was to investigate the impact of an active lifestyle on hemodynamic, HRV and oxidative stress parameters in offspring of hypertensive parents.

## Methods

### Study design

The study protocol was approved by the research ethics committee at the Universidade Nove de Julho and performed in accordance with the principles of the Declaration of Helsinki. All 255 participants signed an informed consent form.

**Figure 1:**
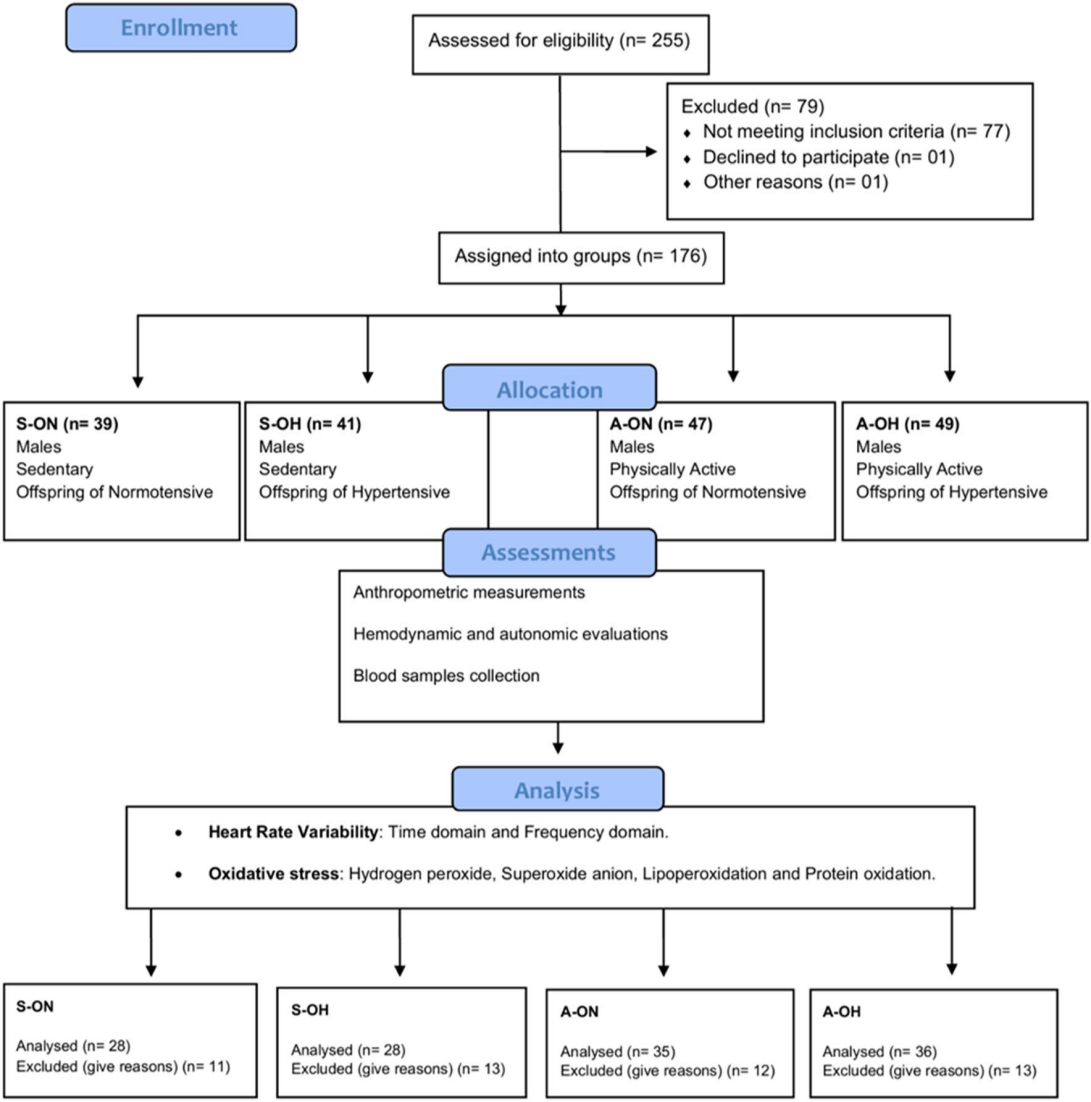
Trial consort flow diagram. International Physical Activity Questionnaire (IPAQ), Anthropometric measurements, Hemodynamic evaluations, HRV analysis. Blood samples collected and oxidative stress assessment.

The 127 participants (recruited from the Military Police of Sao Paulo state) in this study were assigned into four groups: sedentary offspring of normotensive parents (S-ON n = 28); sedentary offspring of hypertensive parents (S-OH n = 28); physically active offspring of normotensive parents (A-ON n = 35); and physically active offspring of hypertensive parents (A-OH n = 36). The screening phase included family and subject medical history, physical examination and anthropometric measures. The trial consort flow diagram is shown in Fig. 1.

Inclusion criteria were as such: Caucasian and non-Caucasian men aged between 18–35 years with BP < 140/ < 90 mmHg, without any serious medical condition and not taking any kind of medication. We excluded subjects having hypertension, severe arrhythmia, a pacemaker, any type of cancer, collagen disease, Crohn’s disease, hypothyroidism or hyperthyroidism, celiac disease and disabling chronic disease, as well as diabetics and subjects with unconfirmed family history of hypertension or with parents or siblings who had diabetes mellitus (type 1 or 2) or secondary forms of hypertension. Therefore, participants who met inclusion/exclusion criteria and completed the screening phase were included in the present study.

The International Physical Activity Questionnaire (IPAQ, version 6) was administered to all subjects in order to establish the level of physical activity as previously described^17^.

### Anthropometric measurements

During the period of the study evaluation, subjects were instructed to avoid alcohol and caffeinated beverages for the preceding 12 h and were invited to come to the laboratory between 08.30 and 12.30 as described elsewhere^17^. On arrival, the subjects underwent routine clinical examination and anthropometric measurements, such as weight, height, and BMI, were obtained while lean mass and percentage body fat were evaluated by bioimpedance (Biodynamics 450). Skinfold thickness measurement was performed using the methodology proposed by Jackson and Pollock^16^.

### Hemodynamic evaluations

As described previously^17^, heart rate (HR), systolic (SBP) and diastolic BP(DBP) were measured at rest in a sitting position. Baseline SBP, DBP and HR were measured three times. Casual BP was measured with a mercury sphygmomanometer, with recommended sized cuffs, by the same researcher. The means of the 3 readings were used for analysis. Heart rate was measured using a heart rate monitor (Polar RS800)^17^.

### HRV analysis

As described previously by our group^17^, the assessment of cardiac autonomic modulation was performed by recording the RR interval using RS800 model of the Polar® heart rate monitor. The RR interval was recorded in the morning for a period of 15 min with the subjects at rest in a sitting position. HRV analysis were made by the average of the three stationary 5 min-series. The series with 50% overlap (Welch Protocol) were divided into continuous segments of 256 data points and were visually inspected for large artifacts or transients that could affect the results. Segments with noisy data were excluded from analysis. RR interval (RR) variability was evaluated in the time and frequency domains. The RMSSD (root mean square of the successive differences of the RR interval) reflects the variability of change in the NN interval. Spectral power for low (LF: 0.03–0.15 Hz) and high (HF: 0.15–0.4 Hz) frequency bands was calculated by means of power spectrum density integration within each frequency bandwidth, using a customized routine (Cardioseries)^17^. The HF band of HRV is considered to reflect the phasic vagal activity triggered by breathing. However, the LF band is more controversial; there are evidences that LF band of HRV is modulated by the sympathetic and the parasympathetic nervous systems as described elsewhere^18,19^.

### Oxidative stress assessment

Plasma samples were prepared from whole blood samples obtained from fasting individuals by venous puncture with heparinized vials for further analysis of oxidant stress profile^20^. Protein was determined by the method of Lowry et al., using bovine serum albumin as the standard as referred elsewhere^21^.

### Hydrogen peroxide

Hydrogen peroxide concentration was assessed based on the horseradish peroxidase – (HRPO) mediated oxidation of phenol red by H_2_O_2_ according with Pick and Keisari^22^.

### Superoxide anion

Superoxide anion was determined in the samples by adrenaline oxidation rate, read in the spectrophotometer at 480 nm as described previously^23^.

### Lipoperoxidation–Thiobarbituric acid reactive substances (TBARS)

As described by Buege and Aust^24^, plasma lipid peroxide levels were determined by measuring TBARS, a common technique for measuring the concentration of malondialdehyde, the main breakdown product of oxidized lipids. For the TBARS assay, using 250 μL of sample, trichloroacetic acid (10%, w/v) was added to the homogenate to precipitate proteins and to acidify the samples. This mixture was then centrifuged (4,000 rpm, 10 min), the protein-free sample was extracted, and thiobarbituric acid (0.67%, w/v) was added to the reaction medium. The tubes were placed in a water bath (100 °C) for 30 min. Absorbencies were measured at 535 nm using a spectrophotometer.

### Protein oxidation (carbonyls)

Protein damage was determined by protein carbonyl measurements, using 200 μL of the sample. Plasma samples were incubated with 2,4-dinitrophenylhydrazine (DNPH 10 mM) in a 2.5 M HCl solution for 1 h at room temperature in the dark. Samples were vortexed every 15 min. Subsequently, a 20% trichloroacetic acid (w/v) solution was added and the solution was incubated on ice for 10 min and centrifuged for 5 min at 1000 g to collect protein precipitates. An additional wash was performed with 10% trichloroacetic acid (w/v). The pellet was washed three times with ethanol/ethyl acetate (1:1) (v/v). The final precipitates were dissolved in 6 M guanidine hydrochloride solution and incubated for 10 min at 37 °C, and the absorbance was measured at 360 nm according with Reznick and Packer^25^.

### Statistical analysis

The power of the sample was calculated a posteriori considering the variances of the groups for the RR variance and the LF/HF obtained a β of 1.0 for both parameters^17^. Data are presented as mean ± SEM. Levene's test was used to assess variance homogeneity. Comparisons between the 4 groups were performed with two-way ANOVA, followed by Bonferroni post hoc test. The association between variables was tested by Pearson correlation. The significance level was set at *p* < 0.05.

## Results

### Active lifestyle induces benefits on HRV

No differences between groups were observed regarding age, weight, height and body mass index, thus confirming the homogeneity of the studied groups. Physical activity increased lean body mass and decreased body fat mass when compared to sedentary groups (Table 1).

**Table 1:** Demographic and anthropometric data of the studied groups.

	S-ON (n = 28)	S-OH (n = 28)	A-ON (n = 35)	A-OH (n = 36)	*P* value
Age (years)	35.0 ± 1.16	36.7 ± 1.25	33.9 ± 0.73	33.4 ± 0.67	0.083
Weight (kg)	84.4 ± 2.22	86.1 ± 2.03	82.1 ± 2.02	81.4 ± 1.96	0.944
Height (cm)	175.5 ± 0.94	176.4 ± 1.23	176.3 ± 1.25	176.3 ± 0.90	0.949
Body mass index (kg/m^2^)	27.6 ± 0.71	27.7 ± 0.48	26.3 ± 0.44	26.1 ± 0.54	0.091
Fat mass (%)	20.9 ± 0.97	22.2 ± 0.83	18.1 ± 0.77§	17.3 ± 0.83*§	0.0001
Lean mass (%)	79.1 ± 0.97	77.8 ± 0.83	81.9 ± 0.77§	82.7 ± 0.83*§	0.0001
SubCut fat mass (mm)	40.8 ± 2.71	42.2 ± 2.23	31.7 ± 2.05*§	28.8 ± 2.13*§	< 0.0001

Values are reported as mean ± SEM. S-ON: sedentary offspring of normotensive parents; S-OH: sedentary offspring of hypertensive parents; A-ON: physically active offspring of normotensive parents and A-OH: physically active offspring of hypertensive parents: SubCut: Subcutaneous. *p < 0.05 versus S-ON. §*p* < 0.05 versus S-OH.

The weekly frequency and minutes spent with physical activity were higher in the A-ON and A-OH groups when compared to sedentary groups (Table 2). However, with regard to risk factors, such as stress levels, smoking and alcohol consumption, no significant difference was observed between groups (Table 2).

**Table 2:** Levels of psychosocial stress and relative frequency of smoking, alcohol consumption and physical activity of studied groups.

	S-ON (n = 28)	S-OH (n = 28)	A-ON (n = 35)	A-OH (n = 36)	*P* value
Psychosocial stress	13.6 ± 0.95	14.3 ± 1.25	15.1 ± 1.05	13.7 ± 1.16	0.781
Smoke (%)	20	12.5	10	7.7	0.452
Alcohol (%)	13.3	18.7	7.5	7.7	0.400
**Physical activity**					
Weekly/frequency	2.0 ± 0.2	2.4 ± 0.3	8.6 ± 0.6*§	9.7 ± 0.6*§	0.001
Minutes/week	126 ± 19.7	131 ± 21.2	675 ± 105.8*§	799 ± 98.4*§	0.001

Values are reported as mean ± SEM. S-ON: sedentary offspring of normotensive parents; S-OH: sedentary offspring of hypertensive parents; A-ON: physically active offspring of normotensive parents and A-OH: physically active offspring of hypertensive parents. *p < 0.05 versus S-ON. §*p* < 0.05 versus S-OH.

**Figure 2:**
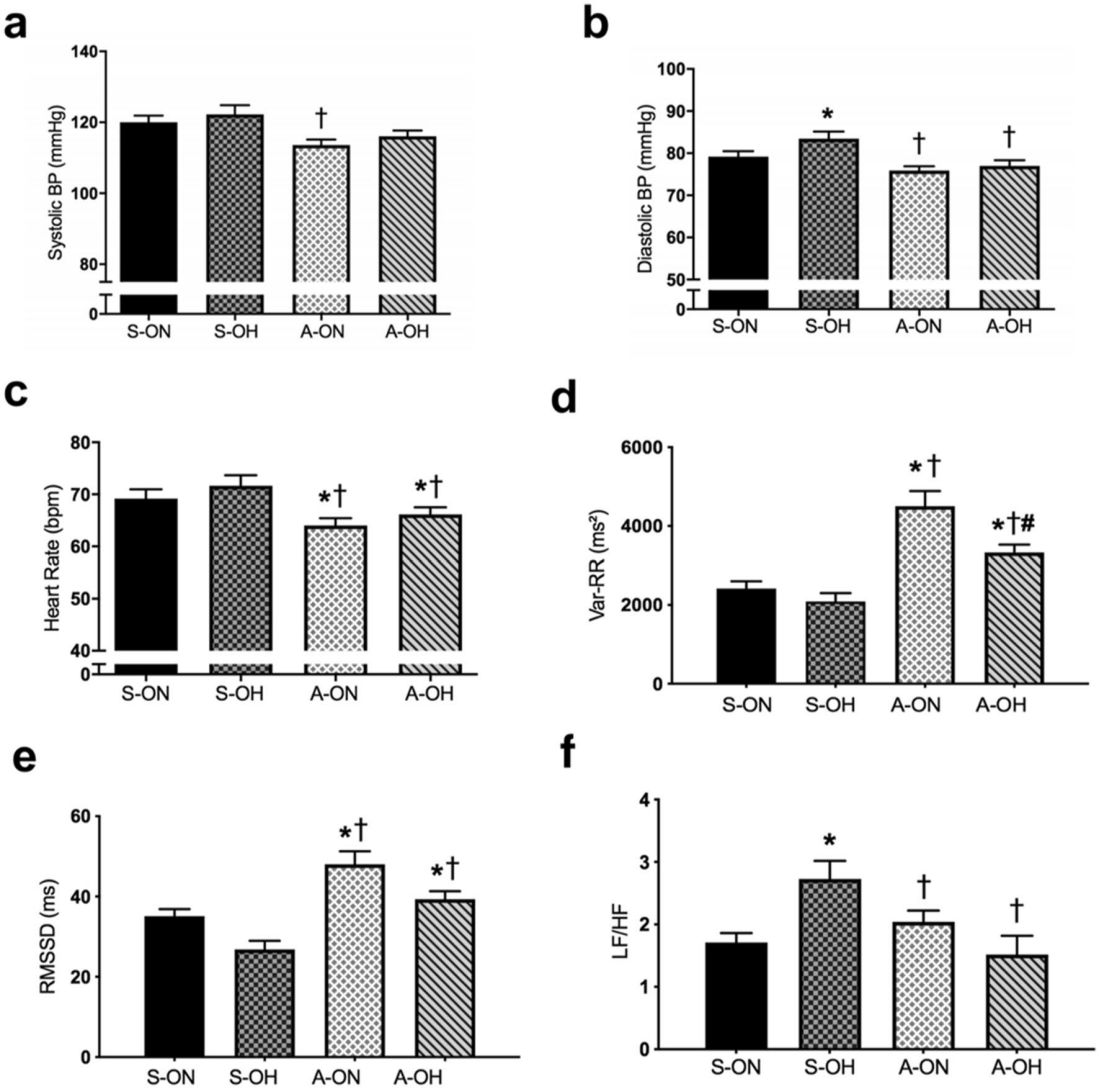
Active lifestyle-induced positive impact on HRV. (**a**) Systolic blood pressure, (**b**) Diastolic blood pressure, (**c**) Heart rate, (**d**) Total variance of RR interval (VAR-RR), (**e**) Root mean square of the successive differences (RMSSD), (**f**) LF/HF ratio. sedentary offspring of normotensive parents (S-ON n = 28); sedentary offspring of hypertensive parents (S-OH n = 28); physically active offspring of normotensive parents (A-ON n = 35); and physically active offspring of hypertensive parents (A-OH n = 36). *p < 0.05 versus S-ON. †*p* < 0.05 versus S-OH. #*p* < 0.05 versus A-ON.

**Figure 3:**
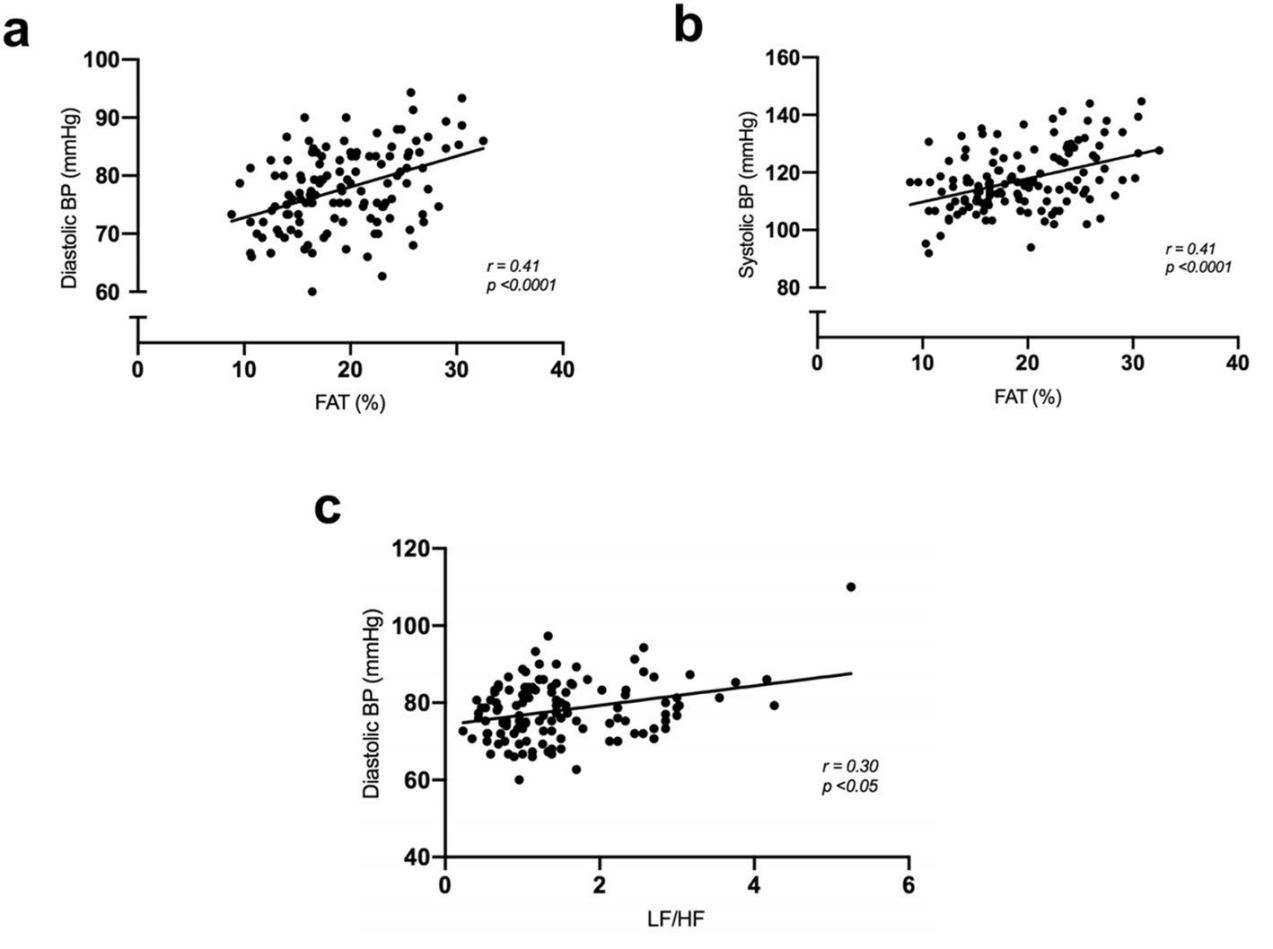
Higher sympathetic modulation and body fat were correlated with higher blood pressure. (**a**) Correlation between percentage of fat mass with diastolic blood pressure, (**b**) Correlation between percentage of fat mass and systolic blood pressure in the studied groups, (**c**) Correlation between LF/HF ratio and diastolic blood pressure.

Sedentary offspring of hypertensives (S-OH) showed increased DBP when compared to offspring of the normotensive group (S-ON). However, physically active groups presented decreased DAP (A-ON and A-OH vs. S-OH). No difference was observed in SBP between the groups (Fig. 2a,b). We observed resting bradycardia (a good indication of overall fitness) in both physically active groups (Fig. 2c). Statistically significant positive correlations were observed between DBP (r = 0.41) and SBP (r = 0.41) (*p* < 0.0001) with the percentage of body fat, demonstrating the great impact of body fat percentage on BP (Fig. 3a,b).

Active lifestyle had a great impact on HRV. Regarding time domain analysis of HRV, physically active groups presented increased values in total RR variance (VAR-RR) (A-ON: 4,912 ± 538 and A-OH: 3,112 ± 236 vs. S-ON: 2,354 ± 159; S-OH: 2,232 ± 241 ms^2^, *p* < 0.0001) and RMSSD when compared to the sedentary ones. However, the values for these parameters remained lowered for the A-OH group when compared to the A-ON group (Fig. 2d,e). In the same way, the physical activity groups (A-ON and A-OH) presented higher values for SDNN when compared to sedentary ones (Fig. S1a).

In frequency domain of HRV measures, the S-OH group presented increased LF nu and decreased HF nu when compared to S-ON group. In contrast, both physically active groups (A-ON and A-OH) showed reduced LF nu and increased HF nu when compared to the S-OH group (Fig. S1b,c). The offspring of hypertensives presented increased LF/HF ratio when compared to SON group (S-OH: 2.02 ± 0.19 vs. S-ON: 1.26 ± 0.08). However, both physically active groups demonstrated a reduction of this parameter (LF/HF, A-ON: 1.25 ± 0.09 and A-OH: 1.17 ± 0.10) (Fig. 2f).

We observed a statistically significant correlation between LF/HF ratio and DBP (r = 0.30, *p* < 0.05) (Fig. 3c), indicating that men presented higher values of LF/HF and demonstrating a higher DBP in the studied population.

### Active lifestyle prevents increase of oxygen reactive species

**Figure 4:**
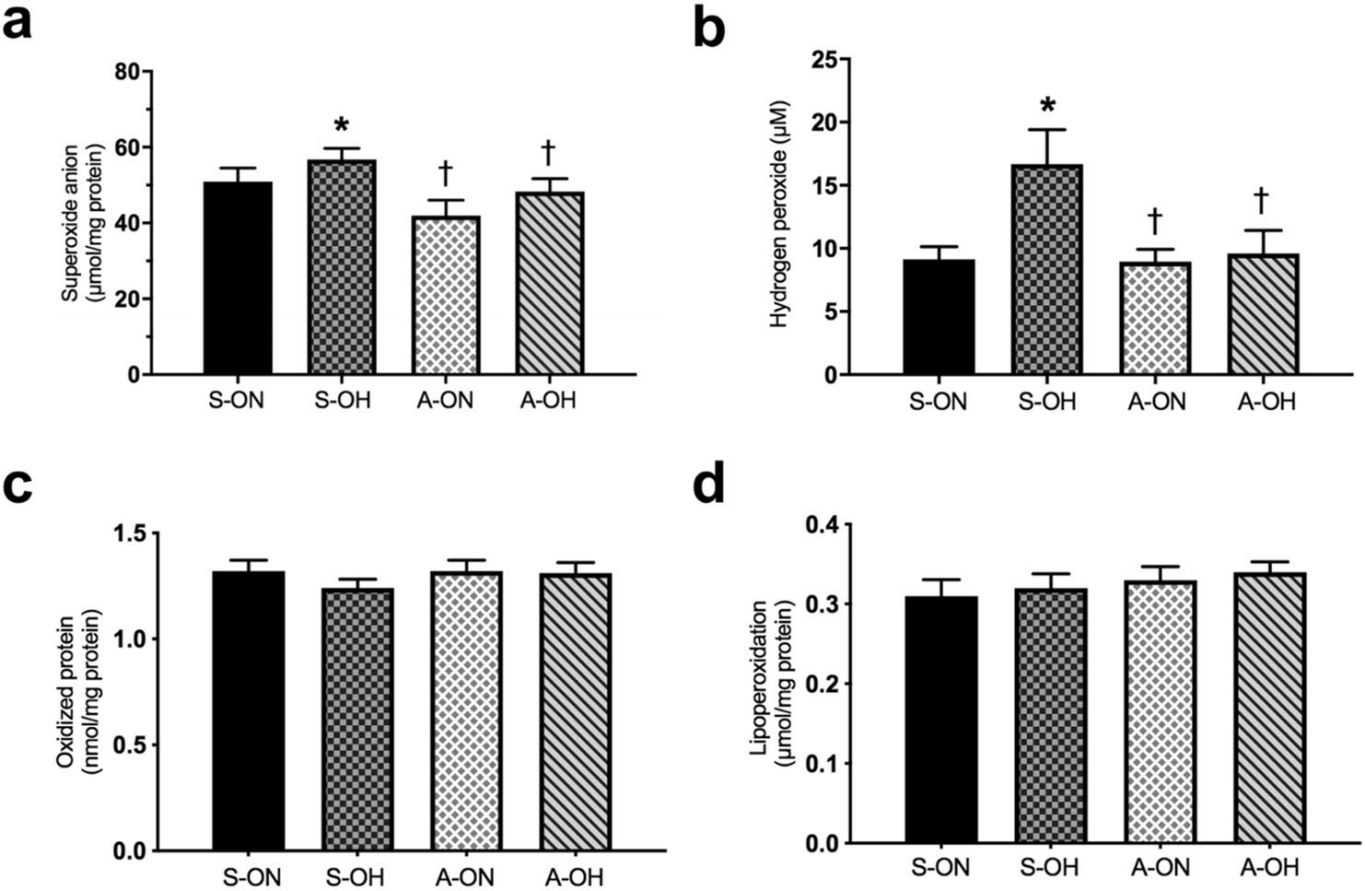
Active lifestyle prevented increase in oxygen reactive species. (**a**) Superoxide anion, (**b**) Hydrogen peroxide, (**c**) Oxidized protein (Carbonyls), (**d**) Lipoperoxidation (TBARS). sedentary offspring of normotensive parents (S-ON n = 28); sedentary offspring of hypertensive parents (S-OH n = 28); physically active offspring of normotensive parents (A-ON n = 35); and physically active offspring of hypertensive parents (A-OH n = 36). *p < 0.05 versus S-ON. †*p* < 0.05 versus S-OH.

**Figure 5:**
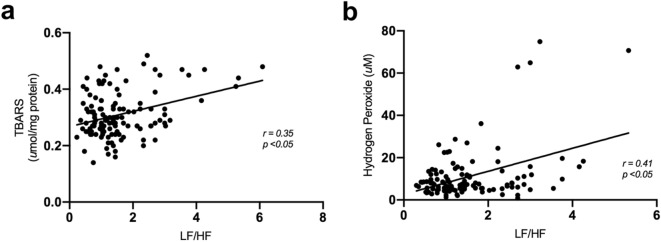
Higher sympathetic modulation was correlated with oxidative stress. (**a**) Correlation between LF/HF ratio and hydrogen peroxide, (**b**) Correlation between LF/HF ratio and lipoperoxidation in the studied groups.

Systemically superoxide anion and hydrogen peroxide were increased in S-OH group when compared to S-ON group. However, both physically active groups (A-ON and A-OH) presented reduced levels of superoxide anion and hydrogen peroxide when compared to S-OH group (Fig. 4a,b).The systemic levels of oxidized protein and lipoperoxidation (assessed by carbonyls and TBARS, respectively) did not present significant differences between the groups (Fig. 4c,d). Statistically significant positive correlations were observed between LF/HF ratio and hydrogen peroxide (r = 0.41, *p* < 0.05), and lipoperoxidation (r = 0.35, *p* < 0.05) (Fig. 5a,b), thus demonstrating that subjects with higher sympathetic modulation presented higher markers of redox imbalance.

## Discussion

To our knowledge, this is the first study reporting the impact of an active lifestyle on hemodynamic, HRV and oxidative stress parameters in offspring of hypertensive parents. Three important findings emerged from the present study. First, the offspring of hypertensive parents showed higher levels of DBP, cardiac sympathetic modulation and systemic reactive oxygen species. Secondly, the higher levels of the cardiac sympathetic modulation were correlated with systemic levels of hydrogen peroxide, lipoperoxidation and DBP. Finally, the major finding of this investigation lies in the fact that an active lifestyle seems to prevent early hemodynamic and HRV dysfunctions, along with oxidative stress in offspring of hypertensive parents.

Most studies on offspring of hypertensives have focused on the autonomic nervous system as a major contributor to the onset of hypertension^11,12^. In order to determine the direct effects of both family history and an active lifestyle on the autonomic nervous system, we selected 127 participants with or without family history of hypertension. For the assessment of the autonomic nervous system we used HRV, an effective tool to obtain reliable indices for overall sympathetic nerve activity and baroreceptor function^10^. Several studies have demonstrated that offspring of hypertensives had higher sympathetic activity^6^ as well as higher rest cardiac sympathetic modulation^13^. However, these autonomic dysfunctions are not necessarily accompanied by clinical symptoms, such as increased BP, as previously described^13,14^. Therefore, the detection of the early autonomic modulation dysfunction, before any clinical manifestation occurs, may have an important role in preventing the onset of hypertension.

In the present study, we also corroborated previously published data showing that offspring of hypertensive parents had autonomic dysfunction^6,13,14^, as demonstrated by higher sympathetic and lower parasympathetic modulation (LF nu and HF nu), thus reflecting in the LF/HF ratio. Pharmacological and non-pharmacological approaches have been studied for the treatment of autonomic dysfunction, prevention of end-target organ damage and the onset of hypertension^15^. Among non-pharmacological strategies, physical activity and/or exercise training has been found to be an effective tool in preventing hypertension^26^.

In our study, the active lifestyle group of offspring of hypertensives presented normalized cardiac autonomic modulation, probably associated with reduced resting HR. These results concur with previous studies involving patients with established hypertension, indicating that an active lifestyle minimizes physiological stress and autonomic alterations^27,28^. Taken together, our findings indicate that activity lifestyle attenuates/prevents the impact of family history on one of the most important and studied factors, i.e., sympathetic modulation, associated with hypertension development.

It should be noted that despite the unfavorable changes in HRV associated with family history of hypertension, hemodynamic values remained within the normal range. However, the sedentary offspring of hypertensive parents showed increased DBP. The physiological mechanisms underlying this increase might be explained by the increase in fat mass and cardiac sympathetic modulation observed in this group. Indeed, changes in the autonomic nervous system over the cardiovascular system tend to occur prior to the increase in BP^29^. We observed a positive correlation between LF/HF ratio and fat mass associated with DBP, indicating that subjects with higher fat mass and cardiac sympathetic modulation presented higher DBP. It is worth mentioning that fat mass has been associated with higher sympathetic tonus and development of hypertension^30^. Additionally, Hesse et al.^31^ have demonstrated that baroreflex sensitivity was inversely correlated with the mean BP evaluated over 24 h and positively associated with HRV. Our results corroborate with these findings, demonstrating that the S-OH group had higher levels of DBP and HRV dysfunction parameters, which occurred in an inverse manner in the physically active groups.

Oxidative stress has been found to play an important role in the development of hypertension and cardiovascular diseases^9,10^. Oxidative stress refers to the imbalance due to excess reactive oxygen species or oxidants over the ability of the cell to build an effective antioxidant response^23^. Hydrogen peroxide is an important ROS in redox signaling^22^. In this sense, although we did observe unchanged systemic levels of markers of oxidative damage, evaluated by lipoperoxidation and protein oxidation, higher levels of systemic hydrogen peroxide and superoxide anion were found in sedentary offspring of hypertensives, suggesting early redox imbalance associated with familial history of hypertension. It should be emphasized that previous studies have demonstrated a positive correlation between cardiac and vascular sympathetic modulation and oxidative stress in experimental model^11,12^. In the present study, we found a positive correlation between LF/HF ratio and hydrogen peroxide and lipoperoxidation, which suggests that increased sympathetic modulation affects oxidative stress profile.

We should like to highlight that only 7 days of fructose overload (10% in drinking water) induced impairment of autonomic control of circulation in spontaneously hypertensive rats (SHR), which is the experimental model more closely related to the essential hypertension, thus showing a strong genetic predisposition to hypertension. Moreover, autonomic changes were followed by unfavorable systemic changes in inflammatory and oxidative stress markers (15–60 days), leading to a later exacerbated increase in BP (only in 60 days) in this model^2,8^. In fact, several studies have associated the autonomic dysfunction on end organ damage with hypertension^15,32^. In this sense, one of the mechanisms thought to be involved in end organ damage is oxidative stress^33^. Therefore, we postulate that the early sympathetic activation in sedentary subjects with family history of hypertension may increase reactive oxygen species, leading to progressive end organ damage and increased BP. Moreover, our data support the hypothesis that an active lifestyle may blunt sympathetic overactivity, body fat accumulation and reactive oxygen species production in offspring of hypertensives, preventing organ damage and BP changes.

Some limitations of the present study need to be addressed to. The first one lies in the use of questionnaires alone to assess the role of family history in hypertension; more extensive information regarding the BP values of parents (normotensive or hypertensive) would be desirable^34^. Secondly, gender limitation may be an issue, since only men have been tested in this trial. Previous studies have shown that women during reproductive life had higher HF band and lower LF band than men^35^, and sedentary lifestyle induces impairment in cardiac autonomic modulation in women^36^. Thus, further studies are needed to determine to what extent gender affects HRV in the offspring of hypertensives. Thirdly, we used an auscultation method for assessing the BP, which is a rather limited procedure when compared to other more comprehensive methods. However, all safeguards have been met ensure the reliability of the final results recorded.

In conclusion, our results lend strong support to the presence of early autonomic dysfunction in offspring of hypertensive parents, which was associated with a systemic increase in reactive oxygen species and blood pressure. However, our most important finding lies in the attenuation of such disorders in physically active offspring of hypertensives, emphasizing the importance of a physically active lifestyle in preventing early dysfunctions potentially associated with the onset of hypertension.

## Supplementary information

Supplementary information
