# Supplementary material for: Impact of an active lifestyle on heart rate variability and oxidative stress markers in offspring of hypertensives
Source: Sci Rep. 2020 Jul 24;10:12439. doi: 10.1038/s41598-020-69104-w (PMC7382460; doi:10.1038/s41598-020-69104-w)

IMPACT OF AN ACTIVE LIFESTYLE ON HEART RATE VARIABILITY AND OXIDATIVE STRESS MARKERS IN OFFSPRING OF HYPERTENSIVES.

F.A. SANTA ROSA, PhD, G.L. SHIMOJO, PhD, D.S. DIAS, PhD, A. VIANA, MSc, F.C. LANZA, PhD, M.C. IRIGOYEN, MD, PhD, K. DE ANGELIS, PhD

Supplementary data

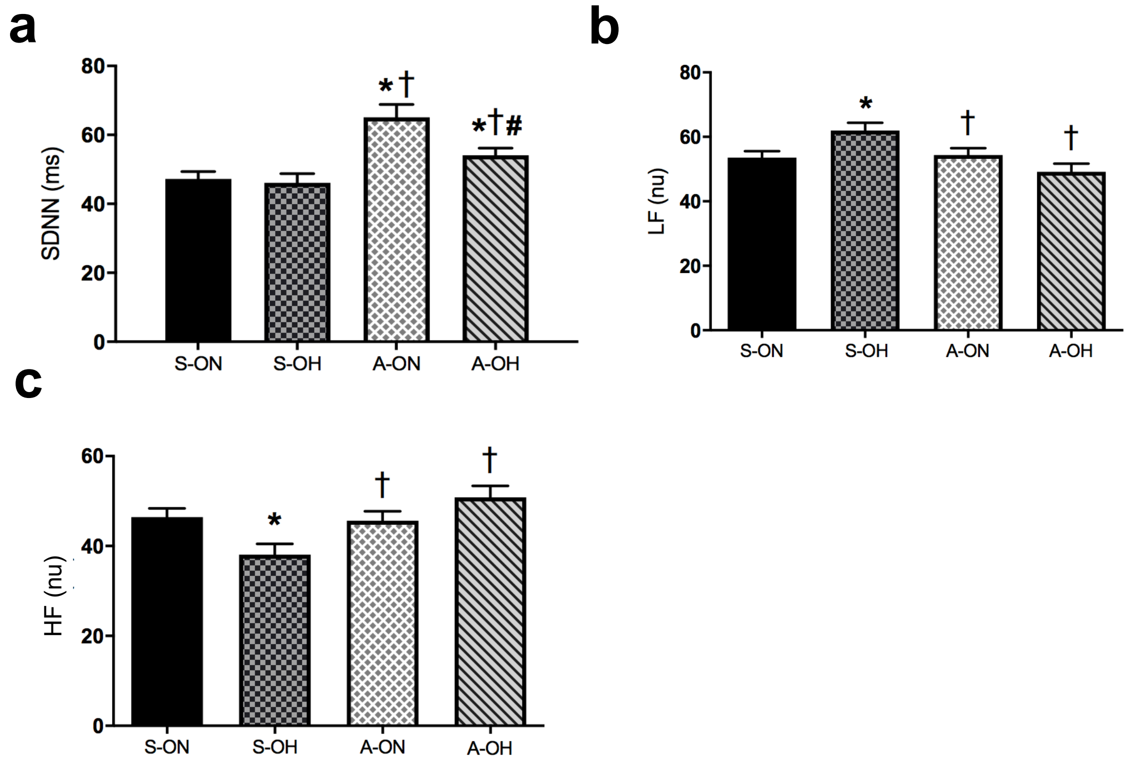

Supplement: Supplementary file 1 — Supplementary information [file 41598_2020_69104_MOESM1_ESM.pdf]
